# TWEAK and Fn14 in the Neurovascular Unit

**DOI:** 10.3389/fimmu.2013.00367

**Published:** 2013-11-11

**Authors:** Manuel Yepes

**Affiliations:** ^1^Department of Neurology and Center for Neurodegenerative Disease, Emory University School of Medicine, Atlanta, GA, USA; ^2^Department of Neurology, Veterans Affairs Medical Center, Emory University School of Medicine, Atlanta, GA, USA

**Keywords:** cerebral ischemia, neurovascular unit, blood-brain barrier, middle cerebral artery occlusion, tumor necrosis factor-like weak inducer of apoptosis, fibroblast growth factor-inducible 14, cerebral edema, nuclear factor kappa-light-chain-enhancer of activated B cells

## Abstract

The neurovascular unit (NVU) is a dynamic structure assembled by endothelial cells (EC), a basement membrane (BM), perivascular astrocytes (PA), pericytes, and surrounding neurons. The NVU regulates the passage of substances and cellular elements from the intravascular space into the brain parenchyma. This function, also known as blood-brain barrier (BBB), is regulated by the integrity of tight junctions proteins between EC, and the interaction between PA and the basal lamina. The cytokine tumor necrosis factor-like weak inducer of apoptosis (TWEAK) and its receptor fibroblast growth factor-inducible 14 (Fn14) are abundantly expressed in the NVU. Here we will review data indicating that the interaction between TWEAK and Fn14 in the endothelial cell-BM-astrocyte interface regulates the function of the BBB following an ischemic/hypoxic injury, and that pharmacological inhibition of TWEAK-Fn14 is a promising target for the treatment of patients with neurological diseases that have a direct impact on the structure and function of the NVU.

## The TWEAK/Fn14 Pathway

Tumor necrosis factor-like weak inducer of apoptosis (TWEAK) is a member of the tumor necrosis factor (TNF) superfamily of cytokines that is synthesized as a type II transmembrane protein from which a soluble ∼17 kDa ligand factor with biological activity can be released [soluble TWEAK (sTWEAK)] (1). The membrane-bound form of TWEAK has an extracellular TNF homology domain (THD) that mediates the assembly of homotrimeric proteins, and a stalk region that contains the recognition site for proteolytic processing by serine proteases of the furin family. TWEAK mRNA is expressed in a variety of tissues and cells, including brain, heart, and lung (2), as well as human endothelial and smooth muscle cells. Soluble TWEAK induces various cellular responses *in vitro*, including cell proliferation (3–5), migration, differentiation (6), angiogenesis (3), and the expression of pro-inflammatory molecules such as IL-8, MCP-1, ICAM-1, and E-selectin in human umbilical endothelial cells (EC) (7), and IL-6, IL-8, and ICAM-1 in astrocytes (8).

Tumor necrosis factor-like weak inducer of apoptosis activity is mediated via binding to fibroblast growth factor-inducible 14 (Fn14), a 14-KDa member of the TNF receptor superfamily (9). Fn14 has a 28 amino acids residues-cytoplasmic tail without a death domain. Instead, Fn14 contains a single binding site for adapter proteins of the TNF receptor-associated factors (TRAF) family (9–11). Fn14 is expressed in a variety of cells and tissue types including tumor cell lines of non-lymphoid origin, fibroblasts, and endothelial and epithelial cells. Furthermore, Fn14 expression is up-regulated following growth factor stimulation of quiescent cell cultures, exposure to hypoxia (12, 13), oxidative stress, chemical and mechanical injuries, inflammation, and tumor growth (14). The TWEAK-Fn14 signal transduction pathway is not fully understood, but it has been shown that TWEAK binding to Fn14 activates the NF-κB (10), extracellular signal-regulated kinase (ERK) (15), and c-Jun NH_2_-terminal kinase (JNK) (15) signal transduction pathways.

In this review we will use the conceptual model of the neurovascular unit (NVU) to discuss the role of TWEAK and Fn14 in the response of the brain to a hypoxic/ischemic injury, and will discuss TWEAK/Fn14 as a potential target for the development of therapeutic strategies to promote cell survival and improve the neurological outcome in patients suffering from acute ischemic stroke and other diseases of the central nervous system (CNS) that affect the NVU.

## The Neurovascular Unit

**Figure 1:**
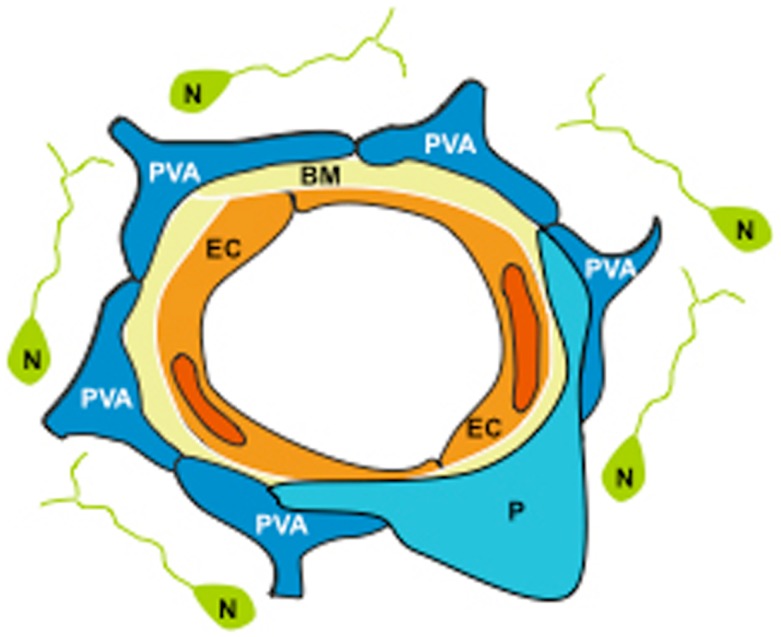
**The neurovascular unit (NVU) is assembled by endothelial cells (EC) ensheated by a basement membrane (BM), embraced by perivascular astrocytes (PVA) and pericytes (P), and surrounded by neurons (N)**.

The NVU is a dynamic structure assembled by EC ensheated by a basal lamina, and surrounded by astrocytic end-feet processes, pericytes, and neurons (Figure 1). One of the main functions of the NVU is to regulate the passage of plasma components and cellular elements from the intravascular space into the brain (16). This barrier function, also known as the blood-brain barrier (BBB), is determined not only by the integrity of the endothelium, but most significantly by a functional interplay between EC, the basal lamina, and perivascular astrocytes (PA).

The permeability of the endothelium is restricted by junctional complexes assembled by adherens and tight junctions proteins (AJ and TJ, respectively). AJ’s are formed mostly by vascular endothelial (VE)-cadherin that mediates cell–cell adhesion via its interaction with the actin cytoskeleton. TJ’s are located in the apical region of the intercellular cleft and function as a “zipper” between the apical and basolateral cell membranes. The transmembrane components of the TJ include junctional adhesion molecule (JAM)-1, occludin, and claudins. A set of accessory proteins, known as zonula occludens (ZO)-1 and ZO-2, link these proteins with the cytoskeleton. Importantly, although disruption of AJ’s may increase the permeability of the NVU, it is primarily the TJ’s that confer the low paracellular permeability and high electrical resistance that characterize the cerebrovascular endothelium.

The basement membrane (BM) forms an interface between EC and PA, pericytes and neurons. It is composed of extracellular matrix proteins such as laminin, collagen IV, fibronectin, and perlecan. About ∼95% of the basal lamina that encases the capillary network is embraced by astrocytic end-feet processes (17), and the opposite pole of each perivascular astrocyte contacts ∼30,000 synapses (18).

The adhesion of EC and astrocytes to the basal lamina requires the interaction of cell-adhesion receptors and their ligands in the matrix. These receptors, known as integrins, play a key role in the activation of cell signaling pathways in response to changes in the extracellular environment, and in the regulation of the interaction between EC and PA. In the NVU the integrin sub-unit β_1_ is found in EC and astrocytes, whereas the integrin α_6_β_4_ is found in astrocytic end-feet processes (19). These receptors interact with laminin-1 and laminin-5 that are found in the basal lamina.

As it will be discussed below, the binding of TWEAK to Fn14 has a direct effect on the composition of the basal lamina and on the interaction between astrocytic end-feet processes and the BM. The interplay between PA and the basal lamina is an important determinant of the permeability of the NVU (20). Indeed, astrocytic-derived factors have a direct effect on the attachment of PA to the BM and on the integrity and distribution of interendothelial TJs proteins, and detachment of astrocytic end-feet processes from the basal lamina result in decreased transendothelial electrical resistance (TEER) across the NVU with increased permeability of the BBB.

Here we will review experimental data indicating that the interaction between TWEAK and Fn14 regulate the interaction between astrocytic end-feet processes and the basal lamina, and that TWEAK/Fn14 play a central role in the regulation of the permeability of the BBB and the survival of neurons associated with each NVU. Furthermore, we will discuss the role of TWEAK and Fn14 in the induction of a pro-inflammatory response in EC and astrocytes, and the infiltration of inflammatory cells from the intravascular space into the brain parenchyma.

## Biological Effects of TWEAK-Fn14 on the Neurovascular Unit

### TWEAK and Fn14 expression in the neurovascular unit

Tumor necrosis factor-like weak inducer of apoptosis and Fn14 are abundantly expressed in the NVU. Indeed, TWEAK and Fn14 have been found in EC of the microcirculation (blood vessels of <100 μm of diameter), in PA, and in neurons of the cerebral cortex, caudate nucleus, putamen, substantia nigra, cerebellar Purkinje cells, and spinal cord (12, 13). Interestingly, *in vivo* studies suggest that the expression of TWEAK and Fn14 in the NVU is cell type-specific. Accordingly, TWEAK appears to be expressed primarily by EC while the highest level of Fn14 expression is detected in PA and neurons. Furthermore, TWEAK has also been found in pericytes (unpublished data), which not only have contractile properties but also secrete several factors, such as angiopoietin-1, that induce the expression of the TJ protein occludin in EC, maintaining the high TEER characteristic of the BBB (21).

### The interaction between TWEAK and Fn14 activates the NF-κB pathway in the neurovascular unit

The NF-κB family includes homo- and heterodimers assembled by the five members of the Rel transcription factor family (22). NF-κB functional complexes are found in EC, PA, and neurons, where they can be activated via two different pathways. In the classic or canonic pathway, IκBα phosphorylation results in the release of NF-κB, leading to its nuclear translocation and DNA binding. In the alternative pathway, IKK1-mediated phosphorylation of the p100 precursor form of the p52 sub-unit leads to proteosomal processing of p100–p52, and nuclear translocation of p52-containing NF-κB dimers (22, 23). Although it has been proposed that TWEAK and Fn14 are able to activate both pathways, *in vitro* and *in vivo* studies suggest that in the brain TWEAK/Fn14-induces NF-κB pathway activation via the canonic pathway. Indeed, incubation of brain microvascular EC, neurons and astrocytes with TWEAK, or the intracerebral injection of TWEAK, leads to IκBα phosphorylation with nuclear translocation of p65 but not p100 phosphorylation (24). However, it has been reported that in other systems such as myoblasts and renal tubular cells, TWEAK may also signal via the non-canonical NF-κB pathway (25, 26). In the brain, treatment with TWEAK induces the expression of ICAM-1, IL-6, and IL-8 in astrocytes (5), and TNF-α in astrocytes and neurons (27). Importantly, the pro-inflammatory effect of TWEAK in the NVU is not limited to astrocytes. Indeed, recent evidence indicates that TWEAK induces an inflammatory response in human cerebral microvascular EC that is associated with increase in the permeability of the BBB (28). Together, these data indicate that the interaction of TWEAK with Fn14 has an NF-κB-mediated pro-inflammatory effect in the different components of the NVU and, as it will be analyzed below, this has a direct effect on the permeability of the BBB, the development of cerebral edema, and neuronal survival.

### Neuronal death

Tumor necrosis factor-like weak inducer of apoptosis can induce cell death in different tumor cell lines via TNF-α-dependent and -independent mechanisms (29). However, this effect is weak and in several experimental systems it requires co-incubation with gamma interferon (1). In contrast, incubation with TWEAK seems to have a stronger effect on neuronal death. Accordingly, treatment of cerebral cortical neurons with TWEAK induces apoptotic cell death (30) and the cleavage and accumulation of poly(ADP-ribose) polymers. This effect is not mediated by TNF-α and instead requires activation of the NF-κB pathway and a functional Fn14 receptor (31). These data suggest that, in contrast with tumor cell lines, the interaction between TWEAK and Fn14 has a direct effect on cell survival in cerebral cortical neurons.

## TWEAK and Fn14 in the Ischemic Brain

### Ischemic stroke

Ischemic stroke is the third cause of mortality a leading cause of disability in the US. About ∼795,000 people suffer a stroke every year, from which 600,000 are first attacks and 195,000 are recurrences (32). Worldwide, it is estimated that 15 million people suffer stroke each year. However, it is important to keep in mind that in many countries ischemic stroke is underreported, and thus this number may be significantly higher. A growing body of experimental evidence in cell cultures (30), rodents (12, 13), and humans (33) indicate that the interaction between TWEAK and Fn14 plays a pivotal role in the response of the brain to an ischemic injury. More importantly, the TWEAK/Fn14 pathway has emerged as a target for the development of pharmacological strategies for the treatment of acute ischemic stroke patients.

### TWEAK and Fn14 in the ischemic brain

Studies with a murine model of cerebral ischemia indicate that the expression of Fn14 mRNA increases in the ischemic tissue as early as 30 min after the occlusion of the middle cerebral artery (hereinafter referred to as MCAO). This effect peaks at 6–24 h, and declines again 48–72 h later. In contrast, the effect on TWEAK mRNA is less well characterized, and while some studies have failed to detect a significant increase in TWEAK mRNA expression in response to the ischemic injury (13), later observations have shown a transient increase in TWEAK mRNA between 30 min and 3 h of the onset of the ischemic injury. Additionally, it has also been reported an effect of cerebral ischemia on TWEAK and Fn14 protein expression. Interestingly, these changes have been detected in the area surrounding the necrotic core, also known as ischemic penumbra, and in those zones of the brain with developing cerebral edema (12, 13). The translational importance of these reports is underscored by recent studies demonstrating that cerebral ischemia also induces the expression of TWEAK and Fn14 in the brain of ischemic stroke patients (33).

### Inhibition of TWEAK – Fn14 interaction has a protective effect in the ischemic brain

The effect of TWEAK/Fn14 inhibition on the outcome of the ischemic injury has been studied with three different strategies: the use of mice genetically deficient on either TWEAK (TWEAK ^−/−^) or Fn14 (Fn14^−/−^), or treatment with either a soluble Fn14-Fc fusion protein, or anti-TWEAK monoclonal antibodies. The first published studies reported that either the intraperitoneal administration of anti-TWEAK monoclonal antibodies 10 min before the induction of cerebral ischemia (30), or the intracerebroventricular injection of an Fn14-Fc decoy receptor immediately after (12, 13, 34) following the onset of ischemic stroke results in a ∼30 and 40% decrease in the volume of the ischemic lesion, respectively. These observations were supported by later studies indicating that when compared to their littermate controls, Fn14^−/−^ mice exhibit ∼60% decrease in the volume of the ischemic lesion following MCAO (34, 35). Additionally, it has been reported that the neuroprotective effect of Fn14^−/−^ deficiency in the ischemic brain is mediated by the induction of the granulocyte-colony stimulating factor (G-CSF) pathway (35). Interestingly, the effect of genetic deficiency of TWEAK on the volume of the ischemic lesion is less dramatic (∼20–40% decrease compared to littermate controls). Together, these data indicate that pharmacological inhibition of TWEAK/Fn14 with Fn14-Fc decoy or TWEAK monoclonal antibodies may be an effective strategy for the treatment of acute ischemic stroke. However, future studies should define if the intravenous administration of these inhibitors also has an effect on the volume of the ischemic lesion, and if so, how far after the onset of the ischemic insult they can be administered.

## The Neurovascular Unit in Ischemic Stroke

Cerebral ischemia has a profound impact on the structure and function of the NVU. Indeed, shortly after the onset of the ischemic injury, those neurons located more distantly from a blood vessel exhibit signs of irreversible injury and death. This is followed by a rapid decrease in the expression of endothelial-β_1_ and astrocytic-β_1_ and -α_6_β_4_ integrins, and degradation of laminin in the BM, redistribution of interendothelial TJs proteins, and increase in the permeability of the NVU, with the sub-sequent development of cerebral edema and hemorrhagic transformation (36, 37). This process is accompanied by the passage of inflammatory cells from the intravascular space into the ischemic tissue where they further increase the permeability of the BBB and have a deleterious effect on the survival of those neurons located more proximally to the blood vessel. As it will be discussed below, the interaction between TWEAK and Fn14 in the NVU plays a pivotal role in the development of cerebral edema, the infiltration of inflammatory cells into the ischemic tissue, and neuronal death. These data indicate that inhibition of TWEAK/Fn14 is an important target for the development of therapeutic strategies aimed at maintaining the structural and functional integrity of the NVU during ischemic stroke.

### TWEAK and Fn14 expression in the neurovascular unit under ischemic conditions

Experimental data with an *in vitro* model of hypoxia show that deprivation of oxygen and glucose increases the expression of TWEAK and Fn14 in astrocytes, EC, and neurons. However, hypoxia has an effect primarily on the expression of TWEAK in EC and Fn14 in PA and neurons (24).

### The interaction between TWEAK and Fn14-induces the passage of inflammatory cells through the endothelial cell-basement membrane-astrocyte interface

NF-κB pathway activation plays a pivotal role in the development of an inflammatory response in the ischemic brain. Earlier studies have demonstrated that TWEAK induces NF-κB activation in EC, astrocytes, and neurons, and that either treatment with an Fn14-Fc decoy, or genetic deficiency of Fn14, abrogates cerebral ischemia-induced Iκκβ and p65 phosphorylation in the ischemic brain (24). Two events of special pathophysiological significance mediated by the NF-κB pathway are the activation of matrix metalloproteinase-9 (MMP-9) in PA, and the passage of inflammatory cells into the ischemic brain parenchyma, both of which have direct effect on the composition of the basal lamina and the permeability of the BBB (see below).

MMP-9 is an NF-κB-regulated pro-inflammatory matrix metalloproteinase that has a direct effect on the composition of the basal lamina (38). MMP-9 expression in the ischemic tissue not only induces proteolytic degradation of the basal lamina but also degradation of TJ proteins, which leads to the development of cerebral edema, hemorrhagic transformation, and cell death. The intracerebral injection of TWEAK induces MMP-9 activation, and either genetic deficiency of Fn14, or treatment with Fn14-Fc decoy inhibits cerebral ischemia-induced MMP-9 activation. Importantly, the effect of Fn14-Fc decoy on MMP-9 is dose-dependent (24).

The monocyte chemoattractant protein-1 (MCP-1) is an NF-κB-regulated cytokine that has chemoattractant properties for monocytes, memory T-lymphocytes, and natural killer cells. Astrocytes are the main source of MCP-1 in the brain. The onset of cerebral ischemia induces a progressive increase in MCP-1 expression in the ischemia tissue, associated with the passage of leukocytes from the intravascular space into the ischemic brain (39). Studies with an *in vitro* model of the BBB and an *in vivo* model of cerebral ischemia indicate that the interaction between astrocytic-derived TWEAK and Fn14-induces the expression of MCP-1 in PA, leading to the recruitment of neutrophils from the intravascular space into the ischemic tissue. This effect, mediated by NF-κB pathway activation, is abrogated by treatment with Fn14-Fc decoy (40). Together, the available data suggest not only that TWEAK plays a central role regulating the process of ischemic inflammation in the brain, but also indicate that TWEAK inhibition with Fn14-Fc decoy may have a role for the treatment of acute ischemic stroke.

### The TWEAK-Fn14 pathway mediates the development of ischemic edema

The structural integrity of the NVU is compromised when the cerebral blood flow falls below 10–15 ml/100 g/min, allowing the passage of fluid, and proteins from the intravascular space into the brain parenchyma (36, 37), with the development of cerebral edema, which is a leading cause of death in the early phases of ischemic stroke (41). Among the most important events underlying the development of ischemic cerebral edema are changes in the composition of the basal lamina with detachment of astrocytic end-feet processes and redistribution of interendothelial TJs proteins (42, 43). A role for TWEAK in the development of cerebral edema was suggested by the observation that the injection of TWEAK directly into the cerebral cortex is followed by detachment of PA from the BM, and the development of areas of focal cerebral edema (24, 34). Sub-sequent studies demonstrated that either genetic deficiency of Fn14 or treatment with Fn14-Fc decoy inhibit MCAO-induced degradation of laminin in the basal lamina (34). Importantly, the effect of TWEAK on the permeability of the NVU in the ischemic brain is mediated by MMP-9 activation (24).

### TWEAK and neuronal death: A double edge sword

#### TWEAK and Fn14 induce neuronal death in the ischemic brain

A growing body of experimental evidence indicates that TWEAK induces neuronal death. Early *in vitro* studies showed that 24 h of incubation with TWEAK induces apoptotic cell death in neurons and that this effect is mediated by NF-κB pathway activation (30). A role for TWEAK on neuronal death in the ischemic brain was later demonstrated by the observation that treatment with either anti-TWEAK neutralizing antibodies (30) or Fn14-Fc decoy (13), or genetic deficiency of Fn14 (34), reduces the volume of the ischemic lesion following MCAO. Sub-sequent studies reported that the interaction between TWEAK and Fn14-induces poly(ADP-ribose) polymerase-1 (PARP-1) activation and that this effect leads to caspase-3 cleavage and apoptotic neuronal death via a TNF-α-independent mechanism (31).

#### TWEAK induces tolerance in the ischemic brain

Ischemic tolerance is an endogenous neuroprotective mechanism whereby exposure to hypoxia/ischemia for a length of time or intensity that is not severe enough to cause cell death (preconditioning event) renders neurons resistant to a sub-sequent lethal hypoxic/ischemic injury (44). The preconditioning event can protect the brain either very soon after its application (early preconditioning) or after a delay of 24–72 h (delayed preconditioning). Studies with an *in vitro* model of hypoxia and an *in vivo* model of cerebral ischemia demonstrated that either treatment with sub-lethal doses of TWEAK, or induction of endogenous of TWEAK and Fn14 expression with sub-lethal hypoxia, renders neurons tolerant to a lethal hypoxic or ischemic injury applied 24 h later. This protective effect is mediated by TWEAK’s ability to induce neuronal TNF-α expression and ERK 1/2 activation, and is mediated by phosphorylation of the B-cell lymphoma 2-associated death promoter protein (Bad) (27).

## Conclusion and Future Directions

In the brain TWEAK and Fn14 play a pivotal role in the response of the NVU an ischemic injury. Accordingly, the interaction between TWEAK and Fn14 in the endothelial cell-BM-astrocyte interface mediates the passage of inflammatory cells into the ischemic tissue. Likewise, *in vivo* and *in vitro* data indicate that TWEAK binding to Fn14 is followed by detachment of astrocytic end-feet processes from the BM, which leads to increase in the permeability of the BBB and cerebral edema. In neurons, TWEAK and Fn14 have a dual role. Hence, while the interaction between TWEAK and Fn14 during the acute stages of the ischemic injury induces neuronal death, exposure to TWEAK at doses that do not produce cell death, renders neurons resistant against a sub-sequent hypoxic/ischemic injury (ischemic tolerance). Taken together, the data published to this data indicate that TWEAK/Fn14 inhibitors may play a role in the treatment of neurological conditions associated with increase in the permeability of the BBB and hypoxia/ischemia-induced neuronal death. The role of TWEAK as an inductor of ischemic tolerance is intriguing and future studies should define whether this cytokine has a role protecting the brain of patients at high risk of ischemic stroke.

## Conflict of Interest Statement

The author declares that the research was conducted in the absence of any commercial or financial relationships that could be construed as a potential conflict of interest.
